# Face the Challenge—Generalization of Presentation Attack Detection

**DOI:** 10.3390/s25185792

**Published:** 2025-09-17

**Authors:** Adam Baran, Ewelina Bartuzi-Trokielewicz

**Affiliations:** 1 Department of Audiovisual Analysis and Biometric Systems, NASK—National Research Institute, 01-045 Warsaw, Poland; 2 Institute of Control and Computation Engineering, Warsaw University of Technology, 00-665 Warsaw, Poland

**Keywords:** biometrics, presentation attacks, face presentation attack detection

## Abstract

Face recognition is one of the most widely adopted biometric technologies, with applications in mobile devices, banking, and access control. However, its widespread use raises security concerns. One of the most common threats is presentation attacks (PAs), in which adversaries spoof the system using printed photos, videos, or masks. Developing effective Presentation Attack Detection (PAD) methods has become critical, yet generalizing to unseen Presentation Attack Instruments (PAIs) remains a major challenge. This is further complicated by the fact that most public PAD datasets are closed and limited in attack diversity and acquisition conditions. Standard evaluation protocols are typically based on intra- and inter-dataset setups, which may not reflect real-world variability. To address this, we propose analyzing presentation attacks using a novel metric, the Presentation Attack Similarity Index, which quantifies the similarity between different attacks. Based on this, we identify Presentation Attack Similarity Clusters, grouping attacks with high interchangeability. This approach offers deeper insight into PAI relationships, allowing for the strategic selection of representative attacks and the design of more balanced training datasets.

## 1. Introduction

Biometric technologies have become an integral part of modern security systems, with face recognition standing out as one of the most widely deployed methods. Its applications span a broad range, from authentication on mobile devices to financial transaction verification and access control. The widespread adoption of face recognition can be attributed to its convenience, speed, and non-invasive nature.

However, despite these advantages, the large-scale deployment of face recognition systems has revealed critical security challenges. One of the most significant threats is the risk of presentation attacks, where adversaries attempt to deceive the system using printed photos, video recreations, or advanced 3D masks. To counteract these threats, Presentation Attack Detection systems have been developed as essential components of biometric security frameworks. Their primary objective is to reliably distinguish genuine authentication attempts (bona fide presentations) from attack presentations.

While existing PAD systems often demonstrate high performance in detecting known attack types, their ability to generalize unseen attack remains a significant challenge. This problem arises due to the wide range of Presentation Attack Instruments (PAIs) that may differ, for example, in texture, material, or resolution, making generalization a key factor in real-world applications.

Moreover, the development of PAD methods that are robust to real-world variability is further hindered by the limitations of existing datasets, which often exhibit limited diversity in attack types and acquisition conditions. These limitations have been recognized, and in response, evaluation protocols for testing the generalization capabilities of PAD methods have been introduced, including intra-dataset, cross-dataset, and Leave-One-Out (LOO) scenarios. Among them, the LOO protocol is particularly challenging, as it evaluates the ability of a PAD method to generalize to entirely unknown datasets or unseen attacks, thus better reflecting real-world deployment conditions.

However, the LOO protocol provides only a general evaluation of the PAD methods’ ability to generalize and does not allow for a more detailed analysis of presentation attacks. Therefore, for a comprehensive assessment of the generalization capabilities of PAD methods, it is necessary to complement existing evaluation methods with a more detailed testing scenario. In this work, we attempt to gain a better understanding of the relationships between different attacks and their impact on the generalization ability of PAD models to unseen attacks. Specifically, this work provides the following:

- A detailed analysis of the impact of presentation attacks on the generalization ability of PAD methods, considering the diversity of attack instruments and presentation styles. To better investigate this, we introduce a new evaluation protocol called Leave-One-PAI-In, in which the model is trained on authentic bona fide samples and a single selected presentation attack scenario, and then tested on all remaining previously unseen attack scenarios. This setup enables a rigorous and informative evaluation of the model’s robustness to diverse and previously unseen attack presentations, providing insights into the ability of PAD models to learn generalizable features from minimal exposure to attack data.
- The definition of a new metric, the Presentation Attack Similarity Index (PASI), which quantifies the similarity between different presentation attacks. The proposed metric can be effectively used to identify gaps in PAD-training datasets, enabling more informed dataset design and improving the generalization capability of PAD methods under real-world conditions.
- The identification of Presentation Attack Similarity Clusters, which group attacks with high feature interchangeability. This clustering provides valuable insights into the relationships between different presentation attack scenarios, supporting the selection of representative attacks to build more balanced and robust training datasets.

## 2. Related Work

Face presentation attack detection has been extensively studied, resulting in numerous proposed methods, with the majority focusing on analyzing image quality degradation. Since PAIs often result in noticeable image quality reduction during the re-capture process, techniques such as Local Binary Patterns (LBPs) [1,2], Histogram of Oriented Gradients (HOG) [3], Difference of Gaussians (DoG) [4], image quality analysis [5], and image distortion analysis [6] have been widely utilized. Additionally, the use of dynamic features extracted from video footage has been explored [7,8,9,10]. However, this approach requires active user cooperation, which significantly limits both the practicality and effectiveness of face recognition systems. Currently, most modern PAD methods rely on the use of convolutional neural networks (CNNs) [11,12]. Among the approaches used are CNN-based models employing stream fusion [13], multichannel architectures [14], pixel-based supervision [15], self-supervised learning [16], attention models [17], and transformer models [18,19].

Although these methods demonstrate strong performance in controlled environments, their effectiveness significantly declines when tested under more realistic or independent conditions. This highlights the limited generalization capability of current PAD models, which remains one of the central and unsolved challenges in the field.

The limited generalization performance is largely attributed to insufficient diversity in training data, the lack of representative attack scenarios, and inconsistencies in evaluation protocols [20]. In response to these limitations, a variety of approaches have been proposed in the literature to improve model robustness, including domain adaptation [21,22], reformulating the PAD task as an anomaly detection problem [20,23], and learning more discriminative feature representations [24].

To reliably assess the generalization ability of PAD models, recent studies increasingly adopt more demanding evaluation protocols, such as cross-dataset testing and modified versions of the Leave-One-Out strategy, including Leave-One-Dataset-Out [25,26] and Leave-One-Attack-Out [14,27,28]. These protocols better reflect real-world deployment scenarios and provide a more accurate assessment of how PAD systems perform under previously unseen attacks and acquisition conditions.

However, it should be noted that even these advanced evaluation procedures do not offer a complete assessment of real-world performance, as they provide only a coarse approximation of the generalization capability of PAD methods.

## 3. Proposed Approach

To systematically evaluate how different PAIs contribute to generalization, we propose a new evaluation approach complemented by additional analytical methods. The goal is to better understand the relationship between PAIs and their impact on the effectiveness of detecting previously unseen attacks. Specifically, we introduce the Leave-One-PAI-In testing protocol and a similarity-based analysis of presentation attacks, which includes the definition of the Presentation Attack Similarity Index and the derivation of Presentation Attack Similarity Clusters.

### 3.1. Leave-One-PAI-In Evaluation Protocol

The goal of the proposed approach is to develop a method for evaluating the generalization capability of PAD model architectures based on minimal knowledge of presentation attacks. To this end, we introduce a new evaluation protocol called Leave-One-PAI-In, in which the model is trained on authentic (bona fide) samples and a single selected Presentation Attack Instrument under a specific presentation scenario.

**Figure 1:**
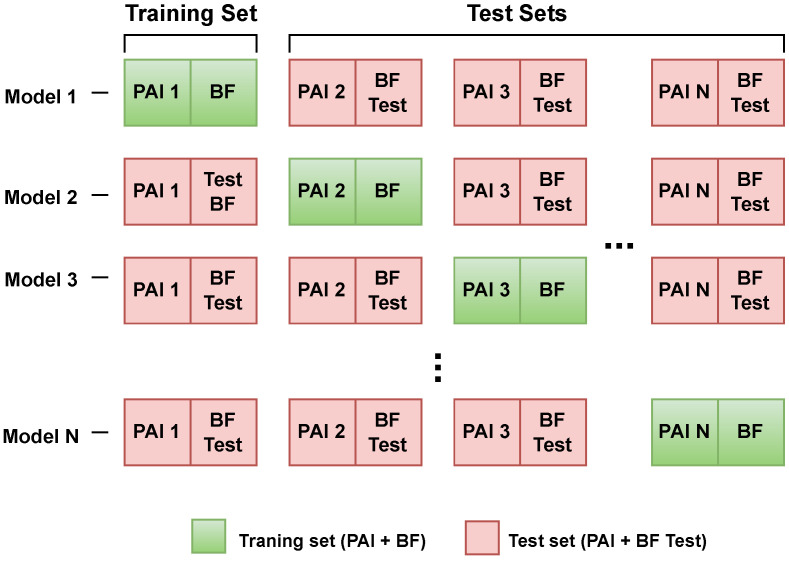
The Leave-One-PAI-In protocol. Models are trained, validated, and tested on bona fide (BF) samples and a single PAI type. Subsequently, the models are tested on all remaining PAI types and bona fide (BF Test) samples to evaluate their generalization capabilities.

The model is then tested on new bona fide samples as well as on each of the remaining PAIs that were not used during training (Figure 1).

This procedure is repeated separately for each PAI, allowing for a detailed analysis of how a particular attack type influences the model’s generalization ability and robustness to diverse presentation attack scenarios. The key metric in this study is the Equal Error Rate (EER), which provides a balanced measure of the overall performance of the PAD method under evaluation. For each PAI, all EER values obtained from testing on the remaining attack scenarios are reported. Subsequently, we compute the following:

- The mean EER for the selected PAI, which indicates how the given attack influences the generalization capacity of the model;
- The Overall Mean EER, defined as the average of all per-PAI mean EERs, allowing for a comparative assessment of the overall generalization ability across different model architectures.

This protocol offers valuable insight into the capacity of PAD models to learn transferable and generalizable features from minimal exposure to attack data. By training a model on a single PAI and evaluating its performance on all remaining PAIs, we assess the extent to which the learned representations capture the fundamental characteristics of presentation attacks, rather than overfitting to specific artifacts.

Furthermore, the variability in generalization performance based on the selected training PAI allows for the identification of both representative and non-representative attack types. PAIs that result in lower mean EERs across unseen attacks may serve as strong candidates for building compact and resilient training datasets. In contrast, PAIs that lead to weaker generalization may indicate the need for broader or semantically richer training data.

Thus, the Leave-One-PAI-In protocol not only benchmarks model robustness under constrained training conditions but also enables a deeper understanding of inter-PAI similarity and its implications for generalization performance.

### 3.2. Presentation Attack Similarity Analysis

Building upon the results obtained from the Leave-One-PAI-In evaluation protocol, we propose a two-step similarity analysis to better understand the interrelationships between presentation attack scenarios and their influence on PAD model generalization.

First, we introduce the Presentation Attack Similarity Index (PASI), which is a metric that quantifies the similarity between attack scenarios based on the model’s classification performance. PASI is computed by measuring the Pearson correlation coefficient between vectors of Equal Error Rates collected in the Leave-One-PAI-In protocol. We used Pearson’s correlation because it measures the similarity in the variability of EER values across attacks rather than their absolute error levels, and it is simple and easy to interpret. Distance-based measures, such as Euclidean distance, would treat such scenarios as far apart even though their actual impact on the model is similar. Negative PASI values indicate that two attacks produce opposing error patterns, while positive values indicate similarity in how the PAD model generalizes to different attacks based solely on limited training data. A high PASI value suggests that two attacks are perceived similarly by the model and elicit comparable generalization behavior.

Second, based on the PASI matrix, we apply agglomerative hierarchical clustering to identify Presentation Attack Similarity Clusters, which are groups of attacks with high mutual interchangeability in terms of their impact on model generalization. The clustering algorithm begins by treating each PASI observation as an individual cluster and iteratively merges the two clusters with the smallest distance, as defined by the Farthest Point Algorithm. In this approach, the distance between clusters *u* and *v* is defined as follows:

(1) $$\begin{array}{c} d(u,v)=\max(\mathrm{dist}(u[i],v[j])) \end{array}$$

where *i* and *j* denote individual attack scenarios within clusters *u* and *v*, respectively. This method promotes the formation of compact, well-separated clusters by ensuring that even the most distant points in a merged cluster remain relatively close to each other.

The clustering process continues until all points are merged into a single hierarchical structure. The resulting dendrogram, visualized in Section 5.3, reveals the nested grouping and similarity structure among the analyzed attack types.

This hierarchical approach allows for the identification of natural groupings of attacks that exhibit similar characteristics from the perspective of PAD models. The resulting clusters offer a structured understanding of which attacks are more representative and how various PAIs relate to each other, ultimately providing a practical tool for constructing balanced and diverse training datasets.

## 4. Experimental Setup

### 4.1. Datasets

In this study, we used the Flickr-PAD [25], FFHQ [29], and OULU-NPU [28] datasets. The Flickr-PAD dataset is a high-quality collection of presentation attack samples captured using various mobile devices.

**Figure 2:**
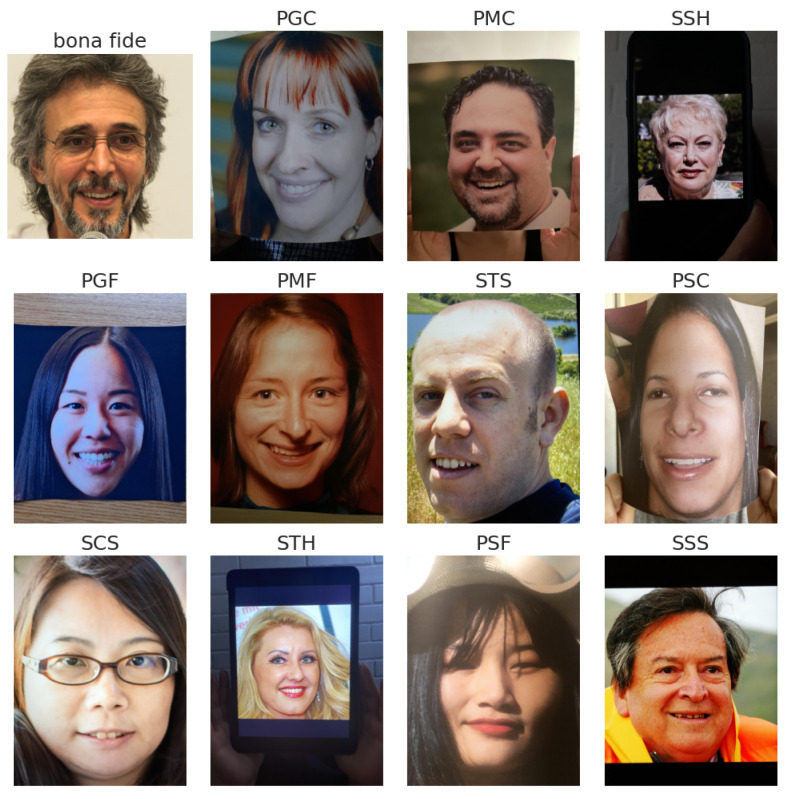
Example images from the Flickr-PAD dataset, including bona fide presentations and various Presentation Attack Instrument (PAI) types, such as printed attacks on different paper types (e.g., glossy, matte, curved) and screen-based attacks captured from smartphones, tablets, and computer monitors under diverse conditions.

It includes 3000 authentic (bona fide) presentations from the FFHQ database and 11,000 PA samples. Figure 2 illustrates the dataset’s diversity, showing bona fide images and a range of PAIs divided into two categories:

- Printed attacks (6000 samples), which are created using various printers (e.g., HP-M479, Epson-2711) and paper types.
- Screen attacks (5000 samples), which consist of photos of images displayed on screens of devices such as laptops, monitors, smartphones, and tablets.

The dataset features 11 distinct types of presentation attack scenarios, making it particularly suitable not only for evaluating the generalization capabilities of PAD models, but also for analyzing relationships and similarities between different types of attacks. Its diversity allows for a more in-depth investigation into how various PAIs influence model performance across different testing conditions. The attack scenarios included are as follows:

- PGC (print–glossy–curved)—printed attacks using glossy paper with a curved surface.
- PGF (print–glossy–flat)—printed attacks using glossy paper on a flat, stable surface.
- PSC (print–std–curved)—printed attacks using standard paper with a curved surface.
- PSF (print–std–flat)—printed attacks using standard paper on a flat, stable surface.
- PMC (print–matte–curved)—printed attacks using matte paper with a curved surface.
- PMF (print–matte–flat)—printed attacks using matte paper on a flat, stable surface.
- SCS (screen–computer–stable)—screen-based attacks using images displayed on a computer monitor, with a stable screen.
- SSS (screen–smartphone–stable)—screen-based attacks using images displayed on a smartphone, fixed during capture.
- SSH (screen–smartphone–held)—screen-based attacks using images displayed on a handheld smartphone.
- STS (screen–tablet–stable)—screen-based attacks using images displayed on a fixed tablet.
- STH (screen–tablet–held)—screen-based attacks using images displayed on a handheld tablet, introducing variability in position and lighting.

In the final experiment, we also used the OULU-NPU dataset, which contains 3960 short video recordings of presentation attacks using printed photos and screens, as well as 990 bona fide videos. For our experiments, we randomly extracted one frame from each video, resulting in a total of 4950 images. The attack types in this database comprise print attacks on glossy paper, produced with two printers (Canon imagePRESS C6011 and Canon PIXMA iX6550), and video-replay attacks displayed on two devices (a 19” Dell UltraSharp monitor and a 13” MacBook Retina).

### 4.2. Baseline Methods

The experiments were conducted using baseline models that represent well-established deep learning architectures, widely used in previous studies on face presentation attack detection.

ResNet [30] is a well-established convolutional neural network known for its robustness and ability to extract deep visual features from images. Characterized by the use of residual connections, which mitigate the vanishing gradient problem and enable effective training of deep networks, it is one of the most popular architectures used in the design of PAD algorithms [13,16,17,18,19,26,31].

MobileNetV3-large [32] is a lightweight convolutional neural network architecture optimized for efficiency and performance. It is designed specifically for real-time applications and resource-constrained environments, such as mobile and embedded devices, and combines depthwise separable convolutions and squeeze-and-excitation layers. A MobileNet-based model was also used in the original Flickr-PAD database paper, supporting its relevance for our evaluation.

DeepPixBiS [15] is a model built on the DenseNet [33] architecture, which efficiently leverages multi-scale feature maps. DenseNet introduces dense connections between layers with matching spatial dimensions, enabling improved gradient flow and implicit deep supervision. The model utilizes the first eight layers (comprising two dense blocks and two transition blocks) from a DenseNet pretrained on ImageNet, resulting in a feature map of size 14×14×384.

The model generates two outputs: a pixel-wise binary map (via a 1×1 convolution followed by a sigmoid activation) and a global binary prediction (via a fully connected layer with sigmoid activation). Both outputs are supervised using Binary Cross Entropy (BCE) loss. The total loss function is defined as the weighted sum of the pixel-wise binary loss and the global binary classification loss:

(2) $$\begin{array}{c} L=\lambda L_{\mathrm{pixel}-\mathrm{wise}-\mathrm{binary}}+\left(1-\lambda \right)L_{\mathrm{binary}} \end{array}$$

where λ is set to 0.5.

MobileViTv2-PAD is a modified version of the MobileViTv2 architecture [34] adapted to the task of face presentation attack detection. MobileViTv2 combines convolutional layers with attention mechanisms, merging the strengths of traditional CNNs and transformers. This hybrid design enhances the model’s ability to capture both local and global features, improving its performance on image recognition tasks while maintaining computational efficiency. MobileViTv2 is particularly promising for PAD tasks where fine-grained feature extraction is critical.

### 4.3. Implementation Details

The experiments were conducted using models pre-trained on ImageNet [35] to leverage their feature extraction capabilities. Training was performed with a batch size of 64 over 30 epochs, starting with an initial learning rate of $1\times 10^{-4}$ adjusted dynamically using the CosineAnnealing [36] learning rate schedule. AdamW’s optimizer [37] was used to ensure efficient training and convergence. The problem of presentation attack detection (PAD) was treated as a binary classification task, where the label “1” represents bona fide samples, and “0” represents attack samples. The ResNet, MobileNetV3-large, and MobileViTv2-PAD models were trained using the Binary Cross Entropy Loss function. In contrast, the DeepPixBiS model was trained with a custom loss function defined as the weighted sum of the pixel-wise binary loss and the global binary classification loss. The best model was selected based on achieving the lowest loss on the validation set.

**Figure 3:**
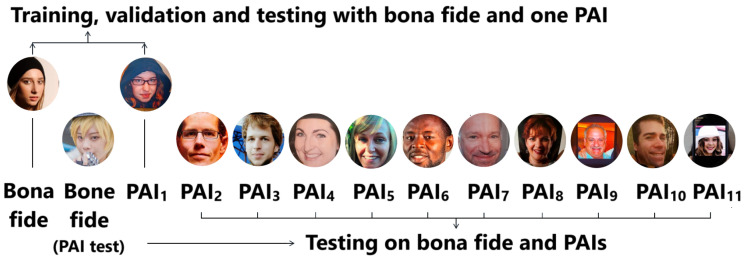
Illustration of the Leave-One-PAI-In protocol applied to the Flickr-PAD dataset.

In the experiments conducted using the Flickr-PAD dataset, the training, validation, and testing processes were carefully designed to evaluate the models in both the Leave-One-Out and Leave-One-PAI-In scenarios:

- Leave-One-Out: 8000 BF samples (oversampled) and 8000 attack samples for training, 1000 BF samples (oversampled) and 1000 attack samples for validation and testing, and 1000 BF samples and 1000 attack samples in test PAI.
- Leave-One-PAI-In: 2400 BF samples and 2400 oversampled attack samples for training, 300 BF samples and 300 oversampled attack samples for validation and testing, and 1000 BF samples and 1000 attack samples in test PAI. The Leave-One-PAI-In protocol for the Flickr-PAD dataset is illustrated in Figure 3.

To ensure high-quality input data for the models, preprocessing steps were applied to all samples. Face detection was performed using a Multi-task Cascaded Convolutional Network (MTCNN) [38], a robust algorithm capable of accurately locating facial regions in images. Following detection, the identified facial regions were cropped and resized to produce square images suitable for model input. Data augmentation was intentionally limited during training to enable precise comparisons of the impact of different PAIs on PAD performance.

### 4.4. Evaluation Metrics

We evaluate the models using ISO/IEC 30107-3 metrics [39] for reporting PAD results:

- Attack Presentation Classification Error Rate (APCER), which is the proportion of attack presentations incorrectly classified as bona fide presentations.
- Bona Fide Presentation Classification Error Rate (BPCER), which is the proportion of bona fide presentations classified as attacks. In addition, we report the operational point BPCER20, which denotes the BPCER value obtained when the APCER is fixed at 5%.
- Equal Error Rate (EER), which is the error rate at the point where where False Acceptance Rate equals False Rejection Rate.

In our experiments, the primary evaluation metric was the Equal Error Rate, since it provides a balanced measure of system performance, with lower EER values corresponding to better performance.

## 5. Experimental Results

### 5.1. Leave-One-Out

In the Leave-One-Out scenario, models were trained on all PAI types except one, which was held out for evaluation. Table 1 reports the obtained EER values, while Table 2 presents the BPCER20 values, which together illustrate the generalization capabilities of ResNet, MobileNetV3-large, DeepPixBiS, and MobileViTv2-PAD when tested on previously unseen PAIs.

The evaluation revealed that printed PAIs, such as print–std–flat and print–std–curved, were among the easiest to detect in the Leave-One-Out scenario, with all models achieving low EERs, even when these attacks were excluded from training. In contrast, screen-based PAIs, particularly screen–tablet–held, screen–tablet–stable and screen–computer–stable, proved significantly more challenging, yielding the highest EERs across all models.

Among the models, MobileViTv2-PAD showed the strongest robustness to unseen PAIs, achieving the lowest EERs on both easy (e.g., 0.7% on print–std–curved) and difficult cases (e.g., 3.3% on screen–tablet–held). This results confirms the advantage of hybrid architectures combining convolutional layers with attention mechanisms, which allows for capturing both local and global contextual patterns. ResNet, MobileNetV3-large, and DeepPixBiS performed comparably on printed attacks but struggled with handheld screen attacks, suggesting that architectural capacity alone is insufficient in the absence of appropriate training diversity. These results confirm that screen-based attacks involving variable lighting and background remain the most difficult to generalize and highlight the advantage of hybrid architectures combining convolution and attention mechanisms.

### 5.2. Leave-One-PAI-In

The results of the experiments conducted using the Flickr-PAD dataset are presented in Table 3. The table presents the mean EERs (%) for all analyzed models trained on one PAI and tested on the remaining attacks. According to the assumptions of the Leave-One-PAI-In protocol, these values reflect the model’s ability to generalize when exposed to only one attack type during training. For each training PAI, the mean EER is calculated to quantify how much a given attack influences the model’s generalization capability.

**Figure 4:**
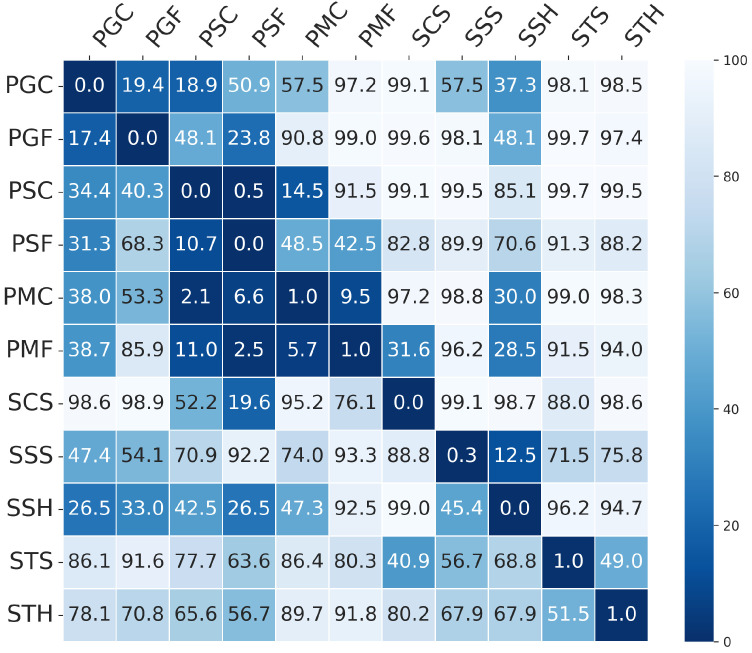
EER (%) results for the Leave-One-PAI-In scenario using MobileViTv2-PAD. The heatmap shows the EER values for models trained on one PAI (row) and tested on other PAIs (columns). Darker shades indicate lower error rates, highlighting the model’s ability to generalize across different PAI types.

Figure 4 provides a more detailed view for the MobileViTv2-PAD model in the form of a heatmap, illustrating EER values obtained in the Leave-One-PAI-In scenario. Rows correspond to the PAIs used for training, while columns represent the PAIs used for testing. Darker shades indicate lower error rates, highlighting which training attacks enable the model to generalize more effectively across different types of presentation attacks.

In line with the purpose of the protocol, PAIs that result in low mean EERs across unseen attack scenarios are considered more representative, as they enable the model to learn generalizable and transferable features. For MobileViTv2-PAD, the best results were observed when the model was trained on the following: print–matte–flat (48.6%), print–matte–curved (53.3%), screen–smartphone–held (60.4%).

In contrast, high EER values indicate challenges in generalizing to new attacks. These issues may arise from overly specific patterns or unique artifacts in the training PAI that do not generalize well to other scenarios. The weakest generalization across all models occurred when training was performed on the following: screen–tablet–held (72.0–97.6%), screen–computer–stable (82.5–92.1%), print–glossy–curved (63.4–91.0%). Such PAIs can be considered less representative, as their inclusion in the training set does not lead to effective recognition of other attack types. Detecting these attacks requires a training dataset that includes sufficient variability and representation of such difficult scenarios.

To compare overall generalization performance between model architectures, we also report the Overall Mean EER, defined as the average of all per-PAI mean EER values. Among the evaluated models, MobileViTv2-PAD demonstrated the strongest generalization performance, achieving the lowest Overall Mean EER of 64.5%, compared to ResNet (81.1%) and MobileNetV3-large (81.3%). MobileViTv2-PAD was effective in both simple and complex attack scenarios, highlighting its ability to learn more robust and transferable features. In contrast, ResNet and MobileNetV3-large achieved good results for selected PAIs but showed limited effectiveness in the presence of more difficult or less representative attacks, such as screen–tablet–held or print–glossy–curved.

In summary, these results confirm the utility of the Leave-One-PAI-In protocol for evaluating model robustness under limited training data and emphasize the importance of selecting representative attack types when constructing compact and effective PAD training datasets.

### 5.3. Clustering of Presentation Attacks Based on PASI

**Figure 5:**
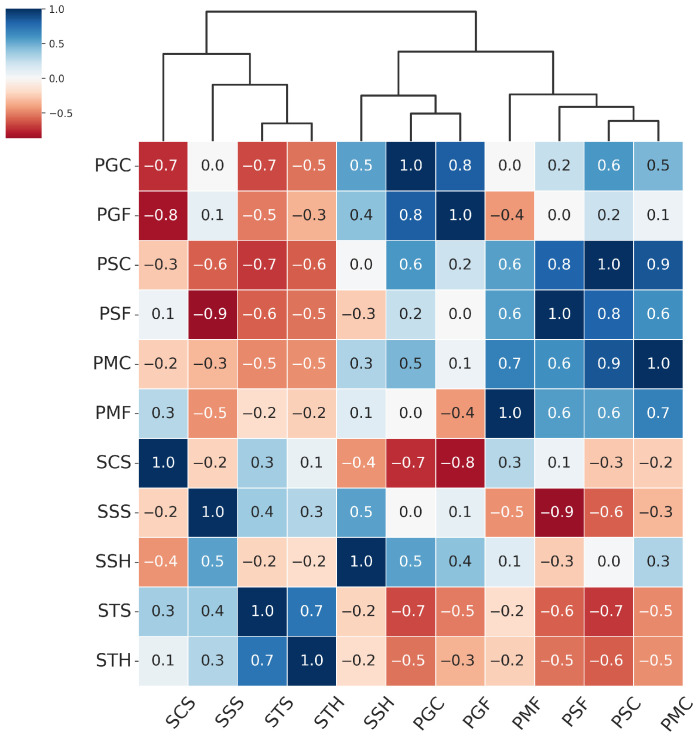
Hierarchically clustered heatmap for PAIS results for MobileViTv2-PAD.

Based on the EER matrix obtained in the Leave-One-PAI-In scenario, we computed a matrix containing Presentation Attack Similarity Index values. This matrix quantifies the degree of similarity between different types of presentation attacks (PAIs) in terms of their impact on the generalization ability of a given PAD model architecture. High PASI values (close to 1) indicate that two attacks are perceived by the model as similar, i.e., they elicit comparable generalization behavior. In contrast, low or negative values suggest a lack of such similarity. The resulting PASI matrix was then used to perform agglomerative hierarchical clustering using the Farthest Point Algorithm, which enabled the identification of groups of attacks with similar effects on model generalization. Importantly, in our study, the number of clusters was not fixed a priori, but derived from the hierarchical dendrogram structure. We focused on cluster stability and interpretability, ensuring that the resulting groups reflected meaningful presentation characteristics such as texture. This approach also allows new PAIs to be dynamically assigned to the existing clusters. While classical validity indices (e.g., silhouette, Davies–Bouldin, Gap Statistic) could also be used, our goal was to derive clusters that are not only statistically consistent but also practically relevant for PAD dataset construction. The results of the PASI analysis and the visualization of the Presentation Attack Similarity Clusters obtained for the MobileViTv2-PAD model, which demonstrated the highest generalization capability, are presented in Figure 5.

From the hierarchical clustering of PAIs, we can distinguish three main groups of attacks: the Paper attacks, the Gloss-based attacks, and the Screen attacks:

- Paper attacks—these include printed attacks on matte and standard materials, covering both flat and curved surfaces. Examples include PMF (print–matte–flat), PSF (print–std–flat), PSC (print–std–curved), PMC (print–matte–curved).
- Gloss-based attacks—reflective attacks involving smartphone screens and glossy prints. For ResNet-50 and MobileNetV3-large, this category includes SSS (screen–smartphone–stable). However, for MobileViTv2, SSS is assigned to the Screen attack category. Other attacks in the Gloss-based category are SSH (screen–smartphone–held), PGC (print–glossy–curved), and PGF (print–glossy–flat). DeepPixBiS, which achieved the weakest performance in this study, assigned only PGC and PGF to this group.
- Screen attacks—these involve tablets and computers in both stable and handheld setups, including STH (screen–tablet–held), SCS (screen–computer–stable), STS (screen–tablet–stable).

**Figure 6:**
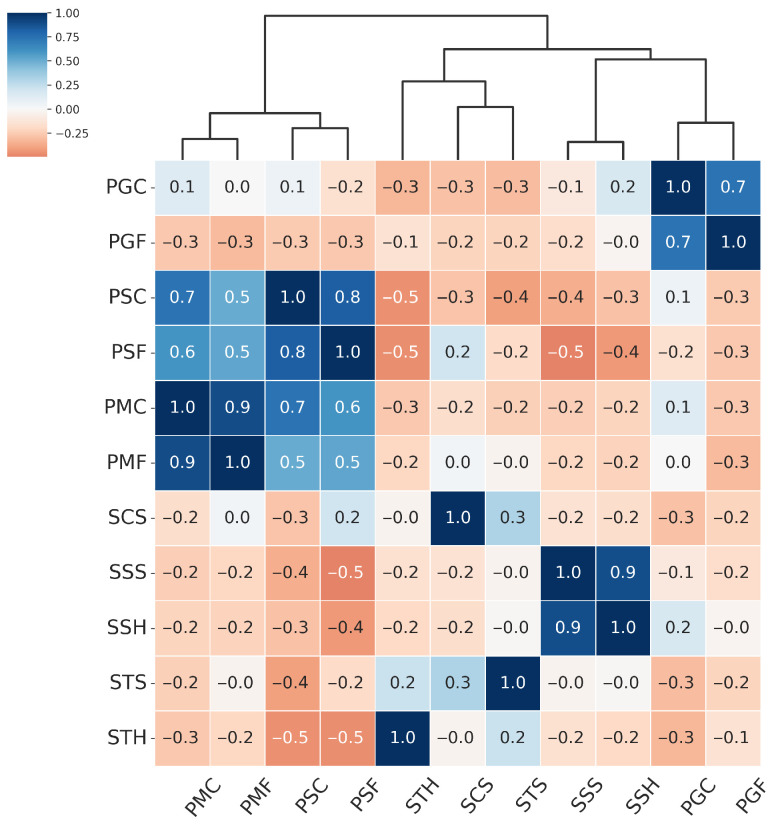
Hierarchically clustered heatmap for PAIS results for MobileNetV3-large.

**Figure 7:**
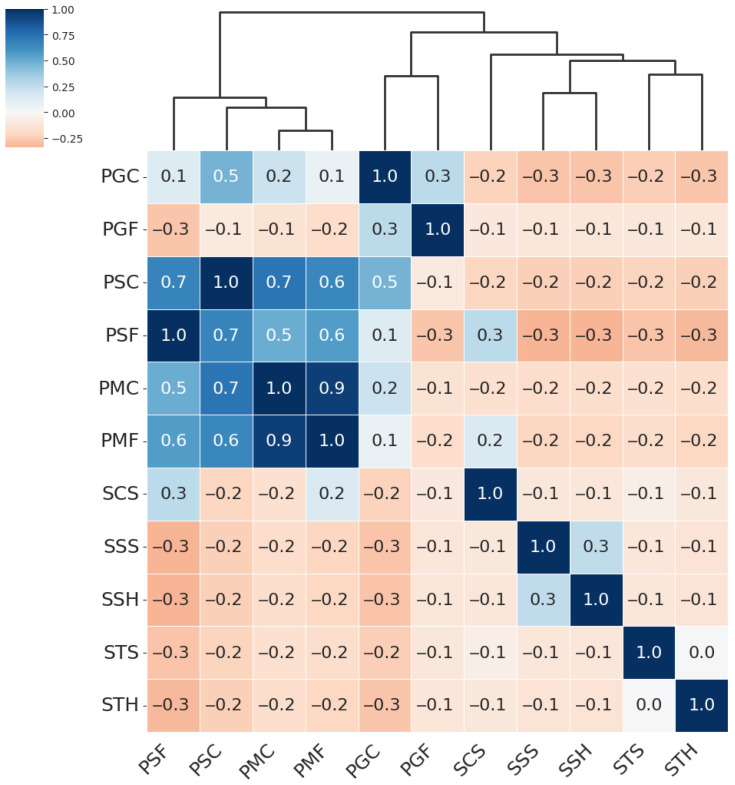
Hierarchically clustered heatmap for PAIS results for DeepPixBiS.

**Figure 8:**
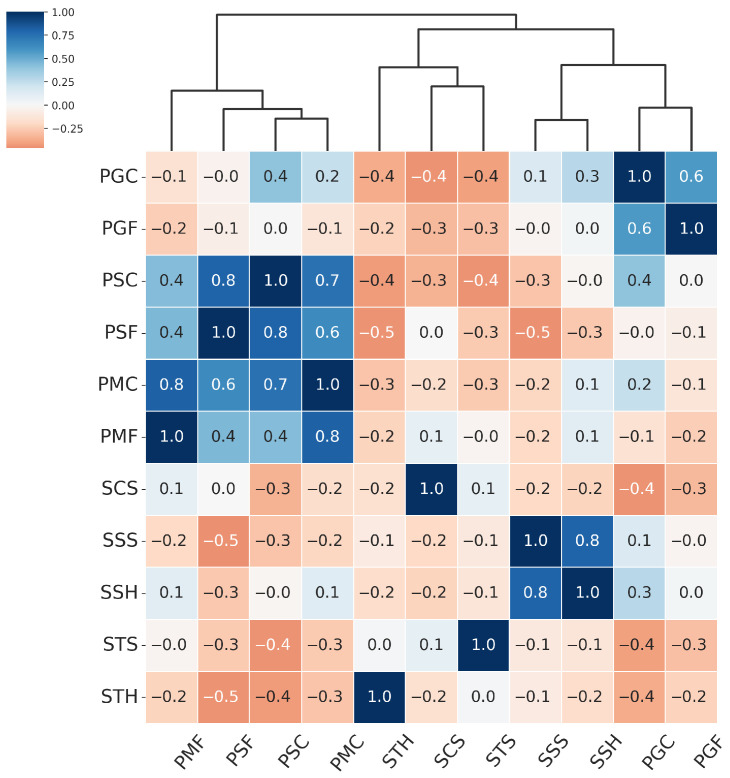
Hierarchically clustered heatmap for PAIS results for Resnet.

These attack categories were independently defined for each of the three evaluated models, as illustrated in Figure 5, Figure 6, Figure 7 and Figure 8. The Paper attacks category, which includes simple paper-based attacks, is identical for all models. The Gloss-based attacks category combines attacks using glossy paper and smartphone screens. In the case of MobileViTv2-PAD, one attack using a smartphone screen has been assigned to the Screen attack category, which focuses on attacks using monitors and tablet screens.

Using insights from the PAI clustering analysis, we conducted an extended version of the Leave-One-PAI-In experiment using the MobileViTv2-PAD model. The objective was to investigate how progressively enriching the training set with representative PAIs from different similarity clusters affects the model’s generalization performance-particularly with respect to previously challenging attacks identified in earlier experiments, such as PSF (print–std–flat), PSC (print–std–curved), STH (screen–tablet–held), and SCS (screen–computer–stable).

In the initial experiment, one representative PAI was selected from each cluster, based on its previously demonstrated high generalization ability (Table 3): Paper attacks—PMF (print–matte–flat); Gloss-based attacks—SSH (screen–smartphone–held); Screen attacks—SSS (screen–smartphone–stable). In the next step, the training set was extended with a second PAI from each cluster: Paper—PMC (print–matte–curved); Gloss-based—PGC (print–glossy–curved); Screen—STS (screen–tablet–stable). In the third experiment, additional PAIs were added to the training set, including those associated with more difficult cases: Paper—PSF (print–std–flat); Gloss-based—PGF (print–glossy–flat); Screen—STH (screen–tablet–held).

In each case, the model was trained on the specified subset of PAIs and evaluated on the remaining attack types, following the Leave-One-PAI-In protocol. The results, summarized in Table 4, indicate that incrementally expanding the training dataset with attacks representing distinct similarity clusters can enhance the model’s generalization performance, particularly for previously difficult scenarios.

**Figure 9:**
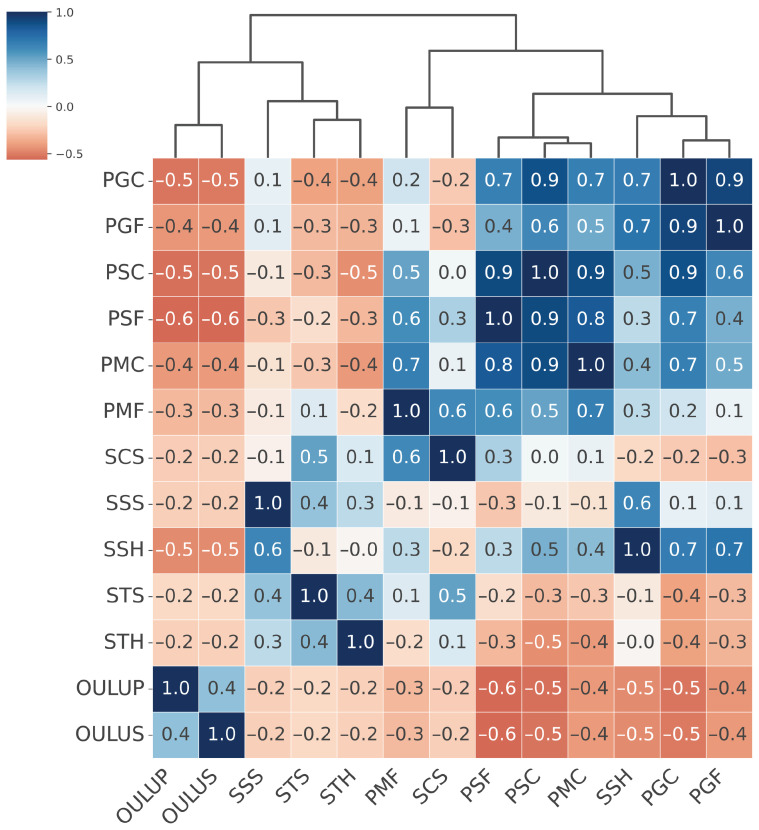
Hierarchically clustered heatmap of PAI results for the MobileViTv2-PAD model on the OULU-NPU and Flickr-PAD datasets.

We also conducted a Leave-One-PAI-In experiment under the Cross-dataset testing protocol. This protocol is particularly demanding, as it evaluates PAD models against previously unseen attacks and under different environmental conditions, resulting from variations in lighting, capture devices, and other factors typical of in-the-wild data. In our experiment, we used the OULU-NPU database, which contains attacks performed with printed photos on glossy paper and video-replay attacks. The print and video-replay attacks were recorded using the front-facing cameras of six different mobile phones. The lighting conditions and acquisition devices differed significantly from those used in the creation of the FFHQ and Flickr-PAD datasets. The results of this experiment are presented in Figure 9.

The attacks from the OULU-NPU database, generated using printed photos on glossy paper and video replay presentations on screens, were found to be most similar to the Screen attacks group, together with SSS (screen–smartphone–stable), STS (screen–tablet–stable), and STH (screen–tablet–held). In the previous experiment with the MobileViTv2-PAD model, the SSS, STS, and STH attacks were also part of this group. At the same time, a clear difference can be observed between the OULU-NPU and Flickr-PAD datasets. The variation in environmental conditions, particularly differences in lighting and the use of distinct attack instruments, demonstrates how strongly dataset characteristics affect model performance in real-world scenarios. The Leave-One-PAI-In experiment under the cross-dataset testing protocol further highlights the need for caution when using external training data collected under conditions that differ from the target deployment environment.

In summary, the PASI-based clustering approach provides a model-driven perspective on presentation attack similarity and can support more informed dataset design by identifying redundant or overly specific attack types that may hinder the generalization ability of PAD models.

## 6. Conclusions

In this study, we introduced a novel evaluation protocol—Leave-One-PAI-In—for assessing the generalization capabilities of face Presentation Attack Detection models under conditions of minimal attack exposure. Unlike standard Leave-One-Out setups, this protocol enables a more fine-grained analysis of how individual PAIs influence model robustness against unseen attacks. Our experiments on the diverse Flickr-PAD dataset revealed substantial variation in generalization performance depending on the training PAI. We found that certain attack types, such as print–matte–flat and screen–smartphone–held, enable stronger generalization and can serve as representative training examples. In contrast, attacks like screen–tablet–held and print–glossy–flat led to poor cross-PAI generalization, suggesting that they introduce overly specific features. To further investigate these differences, we proposed the Presentation Attack Similarity Index and used it to derive Presentation Attack Similarity Clusters via hierarchical clustering. These clusters do not simply reflect the type of instrument used to carry out the attack, but also capture broader presentation characteristics, such as surface texture (e.g., glossy vs. matte), geometric shape (flat vs. curved), and presentation stability (handheld vs. fixed). All of these factors significantly affect how attacks are perceived by the model in terms of generalization behavior. Crucially, the clustering analysis demonstrated that PAD datasets should be constructed in a cluster-aware manner, by explicitly selecting training examples that represent each identified cluster. This ensures that training data include meaningful variation across physical and contextual dimensions of presentation attacks, ultimately improving the model’s robustness to unseen scenarios. For example, in a real-world PAD system, PASI-based analysis could be applied to examine the available training dataset and to derive the Presentation Attack Similarity Clusters. Such an analysis would enable a systematic assessment of the existing dataset and could guide further data collection by deliberately selecting representative attacks from each similarity cluster. This approach reduces redundancy in the data, lowers acquisition costs, and at the same time preserves, or even enhances, the generalization capability of the model.

Overall, our findings highlight the need for dataset design strategies that move beyond attack count and instead emphasize diversity of presentation conditions. The proposed protocol and similarity-driven analysis offer practical tools for constructing compact yet generalizable PAD training sets in real-world conditions.

## Figures and Tables

**Table 1:** EER (%) for Leave-One-Out scenario across models trained on all PAI types except one, evaluated on the excluded PAI. The table presents results for ResNet, MobileNetV3-large (MobileNet), DeepPixBiS, and MobileViTv2-PAD (MobileViT). The “Test set” columns include results for all types of PAIs except the excluded one, which is evaluated separately and presented in the “Unseen PAI” columns.

PAI-Out	ResNet		MobileNet		DeepPixBiS		MobileViT	
	Test Set	Unseen PAI	Test Set	Unseen PAI	Test Set	Unseen PAI	Test Set	Unseen PAI
print–glossy–curved	2.3	1.4	1.4	1.2	1.4	2.1	1.3	1.3
print–glossy–flat	1.5	3.6	2.5	2.8	1.5	4.1	1.4	2.2
print–std–curved	1.0	1.0	2.1	0.9	2.0	1.4	1.3	0.7
print–std–flat	2.4	0.8	1.9	0.6	1.1	1.3	1.0	0.7
print–matte–curved	1.8	2.0	1.8	3.7	2.1	2.7	0.6	2.2
print–matte–flat	2.0	2.9	1.4	2.0	1.8	2.1	0.9	1.8
screen–computer–stable	1.4	5.7	1.0	4.8	1.0	6.7	1.2	4.0
screen–smartphone–stable	0.9	3.2	2.2	4.4	1.1	2.0	1.0	4.2
screen–smartphone–held	1.9	1.9	2.0	1.7	1.1	2.1	1.4	1.3
screen–tablet–stable	1.4	5.5	1.7	4.4	1.6	7.9	1.0	3.9
screen–tablet–held	2.1	7.7	2.1	7.4	0.9	4	0.5	3.3

**Table 2:** BPCER20 (%) for Leave-One-Out scenario across models trained on all PAI types except one, evaluated on the excluded PAI. The table presents results for ResNet, MobileNetV3-large (MobileNet), DeepPixBiS, and MobileViTv2-PAD (MobileViT).

PAI-Out	ResNet		MobileNet		DeepPixBiS		MobileViT	
	Test Set	Unseen PAI	Test Set	Unseen PAI	Test Set	Unseen PAI	Test Set	Unseen PAI
print–glossy–curved	0.0	0.7	0.8	0.9	1.1	1.5	0.0	0.5
print–glossy–flat	0.7	3.6	1.8	1.4	0.0	3.3	0.3	0.9
print–std–curved	0.7	1.1	0.8	0.2	0.3	1.0	0.0	0.2
print–std–flat	1.1	0.4	1.1	0.2	0.4	0.9	0.3	0.3
print–matte–curved	0.7	1.7	1.2	2.8	1.1	1.5	0.0	1.4
print–matte–flat	0.3	2.8	1.1	1.7	0.7	1.5	0.3	1.3
screen–computer–stable	0.0	5.8	0.4	8.0	0.4	8.8	0.0	3.1
screen–smartphone–stable	0.3	2.1	1.2	6.0	0.0	1.0	0.0	3.8
screen–smartphone–held	0.0	1.1	0.4	1.1	0.7	1.3	0.4	0.8
screen–tablet–stable	0.7	5.6	1.2	5.3	1.2	9.7	0.0	3.2
screen–tablet–held	1.2	13.0	0.8	9.6	0.4	4.2	0.0	2.6

**Table 3:** Mean EER (%) for the Leave-One-PAI-In scenario. The table shows the performance of ResNet, MobileNetV3-large (MobileNet), DeepPixBiS, and MobileViTv2-PAD (MobileViT), each trained on a single PAI and tested on all others. The final row presents the Overall Mean EER, reflecting the generalization capability of each model across all PAIs.

PAI-In	ResNet	MobileNet	DeepPixBiS	MobileViT
print–glossy–curved	86.3	91.0	99.8	63.4
print–glossy–flat	85.0	79.5	91.7	72.2
print–std–curved	83.5	85.8	80.8	66.4
print–std–flat	81.0	91.5	99.1	62.4
print–matte–curved	64.4	62.5	63.1	53.3
print–matte–flat	66.4	70.8	75.2	48.6
screen–computer–stable	92.1	83.4	87.5	82.5
screen–smartphone–stable	83.7	89.5	97.8	68.0
screen–smartphone–held	70.0	75.4	97.9	60.4
screen–tablet–stable	91.5	84.8	98.6	70.1
screen–tablet–held	97.6	97.6	99.9	72.0
Overall Mean EER	81.1	81.3	90.18	64.5

**Table 4:** EER (%), APCER (%) and BPCER (%) for the modified Leave-One-PAI-In scenario. The table shows the performance of MobileViTv2-PAD, trained on selected PAIs from each Presentation Attack Similarity Cluster and then tested on all remaining PAIs.

PAI-In	Metric	Test Set	PGC	SSH	PMC	PSF	PSC	STS	STH	SCS
PGF, PMF, SSS	EER	1.0	3.4	1.9	2.8	1.3	1.5	4.6	6.0	4.6
	APCER	0.3	4.3	1.1	3.9	0.7	0.6	9.1	12.3	10.5
	BPCER	1.0	2.2	2.2	2.2	2.2	2.2	2.2	2.2	2.2
PGF, PMF, SSS, PGC, PMC, STS	EER	0.6	-	1.5	-	1.8	0.8	-	4.2	4.0
	APCER	0.6	-	1.1	-	1.1	0.0	-	11.5	8.1
	BPCER	1.3	-	1.9	-	1.9	1.9	-	1.9	1.9
PGF, PMF, SSS, PGC, PMC, STS, PSF, STH	EER	1.0	-	0.9	-	-	0.5	-	-	2.9
	APCER	0.11	-	0.4	-	-	0.1	-	-	3.5
	BPCER	1.66	-	2.4	-	-	2.4	-	-	2.4

## Data Availability

The Flickr-PAD dataset and FFHQ dataset used in this study are publicly available and may be freely used for scientific research purposes.

## Abbreviations

The following abbreviations are used in this manuscript:

PAs	Presentation Attacks
PAD	Presentation Attack Detection
PAIs	Presentation Attack Instruments
PASI	Presentation Attack Similarity Index
EER	Equal Error Rate
PGC	print–glossy–curved
PGF	print–glossy–flat
PSC	print-std-curved
PSF	print–std–flat
PMC	print–matte–curved
PMF	print–matte–flat
SCS	screen-computer-stable
SSS	screen–smartphone–stable
SSH	screen–smartphone–held
STS	screen–tablet–stable
STH	screen–tablet–held
